# Fish Oil-Containing Injectable Lipid Emulsions in Parenteral Nutrition: Immunomodulation and Clinical Outcomes in Critically Ill Patients—Narrative Review

**DOI:** 10.3390/nu18060939

**Published:** 2026-03-17

**Authors:** Mariusz Kęska, Milena Kęska, Mirosław Perliński, Piotr Pabich, Dariusz Onichimowski

**Affiliations:** 1 Department of Anesthesiology and Intensive Care, School of Medicine, Collegium Medicum, University of Warmia and Mazury in Olsztyn, 10-727 Olsztyn, Poland; 2 Clinical Department of Anaesthesiology and Intensive Care, Regional Specialist Hospital in Olsztyn, 10-045 Olsztyn, Poland; 3 Fresenius Kabi Poland, Aleje Jerozolimskie 134, 02-305 Warsaw, Poland; 4 Hospital Pharmacy, Regional Specialist Hospital in Olsztyn, 10-045 Olsztyn, Poland

**Keywords:** parenteral nutrition, fish oil, lipid emulsion, ICU nutrition

## Abstract

Background and Aims: Injectable lipid emulsions are an integral component of parenteral nutrition, providing energy as well as essential fatty acids. However, conventional soybean oil–based emulsions, which are rich in omega-6 fatty acids, are associated with a risk of exacerbating pro-inflammatory responses and immunosuppression, which is of particular importance in critically ill patients. The aim of this review is to present the significance of the composition of modern injectable lipid emulsions, with particular emphasis on emulsions containing fish oil as a source of omega-3 fatty acids (EPA and DHA), and to discuss their potential clinical benefits in selected critical conditions. Methods: This narrative review discusses the rationale for modern mixed-oil ILE, with a focus on fish oil as a source of EPA and DHA, and summarizes potential clinical benefits in selected critical care settings. Results: Modern injectable lipid emulsions combine long-chain triglycerides derived from soybean oil (omega-6), MCTs, olive oil (omega-9), and fish oil (omega-3). Adjusting the supply of individual fractions affects cell membrane structure, signaling pathways, gene expression, and the profile of lipid mediators produced, including specialized pro-resolving mediators (SPMs). ESPEN guidelines and international recommendations emphasize the need to use lipids in parenteral nutrition, preferring mixed-oil ILE supplemented with fish oil. The cited meta-analyses and clinical studies indicate that omega-3-containing emulsions may reduce the risk of infections and sepsis; shorten hospital stay, ICU length of stay, and duration of mechanical ventilation in patients with sepsis; as well as improve outcomes in acute pancreatitis; lower the risk of delirium; and reduce the incidence of delayed gastric emptying. Conclusions: Available data support the use of mixed-oil ILE supplemented with fish oil in the parenteral nutrition of critically ill patients as a strategy with immunomodulatory and pro-resolving potential that may translate into improved clinical outcomes. However, further well-designed randomized trials are needed to optimize dosing and administration regimens.

## 1. Introduction

Since the development of the first-generation total parenteral nutrition (TPN) in the 1960s, injectable lipid emulsions have been a key component, providing both energy and essential fatty acids [1,2]. Early generations of fat emulsions were based primarily on soybean oil, which contains high amounts of omega-6 polyunsaturated fatty acids, particularly linoleic acid as the dominant constituent of this fraction [1]. Over time, however, increased rates of exaggerated inflammatory responses began to be reported in critically ill patients and in individuals receiving long-term parenteral nutrition, and excessive omega-6 fatty-acid delivery was considered a likely contributor to these adverse effects. This hypothesis was supported by the fact that linoleic acid is metabolized to arachidonic acid, a precursor of eicosanoids, including thromboxanes, series-4 leukotrienes, series-2 prostaglandins, and 5-hydroxyeicosatetraenoic acid (5-HETE), which play an important role in regulating inflammation, coagulation, and immune function. These observations stimulated the development of newer-generation injectable lipid emulsions: mixed-lipid formulations with a more balanced fatty-acid profile and potentially more favorable immunometabolic effects. Consequently, emulsions containing not only long-chain triglycerides (LCTs) but medium-chain triglycerides (MCTs), olive oil and fish oil were introduced into clinical practice. Today, changes in injectable lipid emulsion composition are viewed not merely as an adjustment of energy provision but as a form of metabolic intervention that may influence the inflammatory and immune response in critical illness.

## 2. Methodology

This article is a narrative review that aims to synthesize the mechanistic rationale and clinically relevant evidence regarding fish oil-containing injectable lipid emulsions (FO-ILE) used in parenteral nutrition (PN), with a focus on critically ill adult patients. This review integrates concepts related to the fatty-acid composition of modern mixed-oil ILE and their potential influence on inflammation, immunomodulation, and selected clinical outcomes in intensive care.

A targeted literature search was conducted in PubMed/MEDLINE, Embase, Scopus, and the Cochrane Library from inception to November 2025 using combinations of terms related to parenteral nutrition, injectable lipid emulsion, fish oil, omega-3, EPA, DHA, critical illness/ICU, sepsis, infection, length of stay, and mechanical ventilation as well as condition-specific terms reflecting the thematic sections of this review (e.g., acute pancreatitis, COVID-19/ARDS, delirium/CAM-ICU, and gastric emptying/gastroparesis/delayed gastric emptying). In addition, reference lists of relevant clinical trials, meta-analyses, and guideline/consensus documents were screened to identify further key publications.

Because of the narrative design, sources were selected based on relevance, clinical applicability to PN practice, and methodological credibility rather than through a predefined systematic selection protocol. Priority was given to international guidelines, meta-analyses/network meta-analyses, and randomized controlled trials addressing FO-ILE or omega-3-enriched PN in adult critical care. Mechanistic and translational studies were additionally included when they supported the interpretation of clinical findings (e.g., membrane effects, eicosanoid competition, and specialized pro-resolving mediators). The evidence was summarized qualitatively and organized thematically. No formal risk-of-bias tool was applied to each included study, and this review does not aim to be exhaustive; therefore, conclusions emphasize consistency of signals across studies and highlight areas where evidence remains heterogeneous and requires further confirmation in well-designed trials.

## 3. Modern Injectable Lipid Emulsions

At present, injectable lipid emulsions used in parenteral nutrition are mixtures of several lipid fractions, whose proportions determine their biological activity. They typically include the following.

Long-chain triglycerides (LCTs), derived mainly from soybean oil, which are a source of omega-6 fatty acids—primarily linoleic acid. They provide energy and essential fatty acids; however, a limitation is the conversion of linoleic acid into arachidonic acid. Arachidonic acid is a precursor of eicosanoids—compounds that regulate inflammatory responses, immunosuppression, and thrombotic processes. Products derived from arachidonic acid include thromboxanes, series-4 leukotrienes, series-2 prostaglandins, and 5-hydroxyeicosatetraenoic acid (5-HETE) [3].

Medium-chain triglycerides (MCTs)—obtained from refined coconut oil or palm kernel oil—provide a readily available, ketogenic source of energy while not affecting blood triglyceride levels [4,5]. They are characterized by rapid metabolism and a low tendency to be stored as adipose tissue. In addition, compared with pure soybean oil injectable lipid emulsions, they are considered relatively immunoneutral [5].

Olive oil, a source of omega-9 fatty acids, especially oleic acid, which has a smaller impact on immune function, inflammation and blood coagulation than injectable lipid emulsions richer in omega-6 fatty acids [4,6].

Fish oil, a source of omega-3 fatty acids—primarily eicosapentaenoic acid (EPA) and docosahexaenoic acid (DHA)—which exhibit pro-resolving, immunomodulatory, and antioxidant properties.

Differences in emulsion composition translate into differences in biological activity, including effects on cell membranes and multiple regulatory pathways. Fatty acids are incorporated into membrane phospholipids, altering membrane fluidity and micro-organization, which affects the activity of receptors, channels, and signaling complexes. This can lead to changes in the expression of genes related to metabolism, inflammatory responses, and immune function. Particularly important is their influence on the synthesis of bioactive lipid mediators [7].

Injectable lipid emulsions also contain additional components that may be clinically relevant: phytosterols reduce LDL cholesterol absorption; α-tocopherol (vitamin E) has antioxidant properties; and phospholipids—usually phosphatidylcholine—are used as emulsifiers [2,3,4]. This compositional complexity means that the evaluation of injectable lipid emulsions should consider not only the fatty-acid profile but also the potential effects of these additional components.

### 3.1. Beneficial Effects of Omega-3 Fatty Acids: Mechanisms and Biological Rationale

Growing interest in fish oil as part of parenteral nutrition is supported by a growing body of evidence indicating a favorable biological profile of EPA and DHA. Their effects are multifaceted and include modulation of inflammation as well as immune function, hemostasis, and metabolic responses. Consequently, they may reduce the risk of infection and shorten the duration of hospitalization and intensive care unit (ICU) stay [4,6,8].

### 3.2. Competition with Omega-6 Fatty Acids and Modulation of Eicosanoids

Under conditions of increased omega-6 supply—especially when soybean oil predominates—linoleic acid is converted to arachidonic acid, which serves as a substrate for the cyclooxygenase (COX) and lipoxygenase (LOX) pathways. The resulting thromboxanes, series-2 prostaglandins, and series-4 leukotrienes can amplify inflammation, modulate platelet aggregation, and affect the microcirculation. EPA and DHA compete with arachidonic acid for the same enzymes and metabolic pathways, leading to the formation of eicosanoids with a different biological activity profile, i.e., mediators with pro-resolving and immunomodulatory properties. Clinically, this suggests the possibility of shifting lipid-mediator pathways toward a less pro-inflammatory state, which is particularly desirable in patients with sepsis, severe systemic inflammatory responses, and immune dysregulation.

### 3.3. Specialized Pro-Resolving Mediators (SPMs)

A key element in the current understanding of omega-3 mechanisms is the role of specialized pro-resolving mediators (SPMs) [9,10]. EPA and DHA are precursors of resolvins, while DHA additionally gives rise to protectins and maresins. SPMs contribute to the resolution of acute inflammation, limit tissue damage, support clearance of necrotic cells and microorganisms, and facilitate the return to homeostasis [5,9,11,12]. The paradigm of acute inflammation is viewed as a two-stage process: initiation and resolution [13]. In this context, chronicity or an excessively pronounced inflammatory response may result not only from overproduction of pro-inflammatory mediators but also from impaired resolution and insufficient SPM activity. As substrates for SPM synthesis, omega-3 fatty acids may, therefore, serve as a strategy to support the physiological resolution of inflammation rather than merely suppressing it.

### 3.4. Immunomodulation and Inflammatory Markers

Many studies provide evidence that omega-3 fatty acids may reduce the release of pro-inflammatory cytokines and decrease concentrations of inflammatory markers such as CRP and PCT in ICU patients. Meta-analyses have also reported beneficial effects on inflammatory parameters including leukocytosis, IL-1, IL-6, and TNF-α as well as changes in SOFA score dynamics at selected time points. Such findings may serve as a bridge between biological mechanisms and hard clinical endpoints, such as the risk of infectious complications or length of hospital stay.

Omega-3 deficiency may lead to metabolic disturbances, weakened immunity, and increased susceptibility to chronic inflammatory states. Conversely, an excess of omega-6 fatty acids further enhances the synthesis of pro-inflammatory mediators and may disrupt immune balance. For this reason, increasing emphasis is placed on maintaining an appropriate omega-3 to omega-6 ratio in the diet.

### 3.5. Guidelines and Recommendations for Lipid Provision in Parenteral Nutrition

According to the current ESPEN 2023 guidelines for the nutritional management of ICU patients, administration of lipid emulsions is considered an essential component of parenteral nutrition, and it is recommended that lipid intake should not exceed 1.5 g/kg body weight/day and should be adjusted to the patient’s individual tolerance [14]. Excessive dosing may lead to lipid accumulation, metabolic inefficiency, and even toxicity. Particular caution is warranted in patients receiving propofol infusion, as propofol is delivered in a lipid emulsion and constitutes an additional source of fatty acids; at 1.1 kcal/mL, it may substantially increase total energy delivery [15]. The guidelines also suggest using mixed injectable lipid emulsions containing MCTs, omega-9 fatty acids and omega-3 fatty acids.

Similar recommendations are presented in the International Lipids in PN Summit 2022 and the consensus from the meeting of a team of experts with extensive clinical experience in the use of PN [Bydgoszcz, 2024], both aiming to develop practical guidance on the use of injectable lipid emulsions in parenteral nutrition. Both expert groups recognized injectable lipid emulsions as an integral part of parenteral nutrition and additionally recommended that they be based on mixed-oil ILE containing fish oil as a source of EPA and DHA [16,17]. These documents also emphasize the role of omega-3 fatty acids as precursors of specialized pro-resolving mediators (SPMs) and their importance in immunomodulating the inflammatory response.

In recent years, due to growing interest in the immunomodulatory properties of omega-3 fatty acids, numerous studies have been published to confirm their potential clinical benefits. The results of clinical studies have been subjected to numerous meta-analyses. One of these was conducted by Pradelli et al. [18], who performed a network meta-analysis (NMA) to compare and rank different types of injectable lipid emulsions with respect to their effects on infections, sepsis, ICU length of stay, hospital length of stay, and in-hospital mortality. Their analysis included randomized controlled trials evaluating injectable lipid emulsions used in parenteral nutrition in which parenteral nutrition provided at least 70% of total energy intake. The emulsions were classified into four categories: fish oil-containing ILE (FO-ILE), olive oil-based ILE (OO-ILE), medium-chain triglycerides (MCT)/soybean oil (SO)-ILE and pure SO-ILE. After searching the available databases, they ultimately included 47 trials in the NMA, on the basis of which they reported the following results:

- **Infection risk** was reported in 28 trials, including a total of 3081 participants. The network meta-analysis demonstrated very high certainty of a reduction in infection risk with fish oil-based injectable lipid emulsions (FO-ILE) compared with soybean oil-based emulsions (SO-ILE) (OR = 0.43; 90% CrI 0.29–0.63), MCT/SO-ILE (OR = 0.59; 90% CrI 0.43–0.82), and olive oil-based emulsions (OO-ILE) (OR = 0.56; 90% CrI 0.33–0.91).
- **Sepsis risk** was reported in 10 trials, including 1627 patients. The NMA showed a very high-certainty reduction in sepsis risk for FO-ILE compared with SO-ILE (OR = 0.22; 90% CrI 0.08–0.59). OO-ILE also suggested a possible protective effect versus SO-ILE (OR = 0.32; 95% CrI 0.08–1), although with greater uncertainty. No significant differences were found in other comparisons.
- **ICU length of stay (ICU-LOS)** was assessed in 12 trials, including 1163 participants. The NMA found no significant differences between groups, with moderate heterogeneity (I^2^ = 47.8%). Hospital length of stay was analyzed in 28 trials, including 3343 patients. A significant reduction in hospital stay was observed when FO-ILE was compared with SO-ILE (MD = −2.31 days; 90% CrI −3.14 to −1.59) and with MCT/SO-ILE (MD = −2.01 days; 90% CrI −2.82 to −1.22). OO-ILE showed a smaller and less certain effect (MD = −1.46 days; 90% CrI −3.2 to 0.16).
- **In-hospital** mortality was reported in 31 trials, including 2828 participants. FO-ILE showed a possible, high-certainty reduction in mortality versus SO-ILE (OR = 0.67; 90% CrI 0.42–1.06), although the credible interval also included no effect. No other type of emulsion showed a significant impact on survival.
- **Ranking analyses (SUCRA)**: In SUCRA analyses, FO-ILE ranked first across all five assessed endpoints, with the highest probability of being most effective in reducing infection risk (99.0%) and shortening hospitalization (93.2%). OO-ILE ranked second for reducing sepsis risk and shortening ICU and hospital length of stay and third for reducing infections and mortality. SO-ILE consistently ranked last with respect to mortality, infection and sepsis risk, and hospital length of stay. For ICU length of stay, SO/MCT-ILE received the lowest ranking.

Another meta-analysis supporting the beneficial impact of omega-3 use is that presented by Wang et al. [19], which analyzed 25 randomized trials, including 1903 adult patients with sepsis who received nutritional supplementation containing fish oil. Based on this analysis, fish oil supplementation significantly reduced all-cause mortality compared with nutrition without fish oil or omega-3 fatty acids (RR = 0.74; 95% CI 0.63–0.86; *p* < 0.0001). Fish oil also significantly shortened hospital length of stay (MD—9.92 days; 95% CI −15.37 to −4.46; *p* = 0.0004; I^2^ = 91%), ICU length of stay (MD—3.57 days; 95% CI −4.54 to −2.59; *p* < 0.00001; I^2^ = 76%), and duration of mechanical ventilation (MD—2.84 days; 95% CI −5.24 to −0.44; *p* = 0.02; I^2^ = 67%), indicating potential clinical relevance in the treatment of patients with sepsis.

Overall, these findings support the use of fish oil-containing emulsions as an adjunct to nutritional therapy in septic patients, particularly within parenteral nutrition, while highlighting the need for further well-designed RCTs to confirm optimal doses and administration regimens.

Available databases also contain substantial information on other conditions in which omega-3 fatty acids may provide benefits.

### 3.6. Acute Pancreatitis (AP)

Intravenous infusions of omega-3 fatty acids may also be beneficial in patients with predicted severe acute pancreatitis. This was reported by D. Al-Leswas et al. in a randomized, double-blind trial. In the early stage, the disease is characterized by the release of large amounts of pro-inflammatory cytokines, and in approximately 20% of cases, it may progress to severe acute pancreatitis (SAP) [20,21], followed by multiple organ dysfunction syndrome (MODS), which is associated with an approximately 35% mortality risk [22,23].

Currently, standard early management of acute pancreatitis relies primarily on analgesia, fluid resuscitation, and optimal nutritional support. Despite numerous attempts, no approach has been consistently successful in attenuating the pro-inflammatory response and reducing mortality. Increasing evidence suggests positive effects of injectable lipid emulsions providing omega-3 fatty acids, not only because they are an efficient energy source and may reduce carbohydrate requirements but also because they influence the structure and function of cell membranes, the production of signaling molecules, and the regulation of gene expression. Consequently, the fatty-acid composition of injectable lipid emulsions may affect the patient’s metabolic, immune, and inflammatory responses.

In their randomized controlled trial, Al-Leswas et al. [24] enrolled 45 patients, who were divided into two groups: a total of 23 patients received an omega-3-enriched injectable lipid emulsion, and 22 patients in the control group received a standard injectable lipid emulsion. The groups were comparable at baseline, including predicted disease severity and APACHE II scores at admission. Fish oil administration was associated with lower total leukocyte count (*p* = 0.04), CRP (*p* = 0.013), interleukin-8 (*p* = 0.05) and intercellular adhesion molecule-1 (ICAM-1) (*p* = 0.01) as well as improved measures of organ dysfunction, including the SOFA score (*p* = 0.004). The fish oil group also showed fewer new organ failures (*p* = 0.07), a lower rate of admission to critical care, and a shorter overall hospital stay (*p* = 0.04). Based on these findings, the authors concluded that intravenous fish oil-containing injectable lipid emulsions, as a source of omega-3 fatty acids, improve clinical outcomes in patients with predicted SAP, with benefits potentially related to reduced inflammation.

Similar conclusions were reached by Wolbrink et al. [25], who performed a meta-analysis of randomized controlled trials including patients with acute pancreatitis or sepsis treated with omega-3 fatty acids. To assess whether omega-3 supplementation had any beneficial effect, they evaluated CRP and IL-6 at admission and after 7 days. They screened 1332 records and included five trials involving 229 patients. In four studies, including 169 patients with acute pancreatitis, they observed a modest reduction in mortality compared with control groups. Based on two studies (n = 85), omega-3 fatty-acid supplementation was associated with a lower risk of organ failure, and supplementation also showed a trend toward reducing the risk of infectious complications [24,26,27,28].

### 3.7. Delirium/Disorders of Consciousness

Delirium is a serious condition frequently encountered in the intensive care unit (ICU), characterized by an acute deterioration in mental function, including impaired attention, cognitive dysfunction, and an altered level of consciousness. Sepsis-associated delirium (SAD) affects up to 50% of patients and substantially worsens treatment outcomes [29]. Patients with SAD have a markedly higher risk of complications, resulting in prolonged ICU and hospital stays, higher treatment costs, and increased mortality [30]. Although the exact cause of delirium remains unclear, inflammation is considered to play an important role in its development [31,32]. Numerous studies have linked inflammatory processes with delirium, prompting further investigation of pro-resolving therapies aimed at preventing this condition [33,34,35,36].

Based on the well-documented pro-resolving properties of omega-3 fatty acids, which inhibit mediators such as IL-1, IL-6, and TNF-α; increase pro-resolving mediators (resolvins, protectins, maresins); and modulate immune function, Wang et al. evaluated their potential role in preventing delirium in ICU patients with sepsis [37]. Earlier studies had also suggested that omega-3 fatty acids might exert beneficial effects in other neurological disorders [38,39].

In their randomized clinical trial, participants were assigned to two groups. The intervention group received a daily injectable lipid emulsion containing omega-3 fatty acids (fish oil), whereas the control group received a placebo. Patients were assessed twice daily using the RASS and CAM-ICU scales. Ultimately, 207 patients were enrolled and allocated to the two groups: a total of 103 patients received omega-3 infusion, and 104 were assigned to the control group.

The reported findings suggest that omega-3 supplementation may potentially reduce the risk of ICU-associated delirium and improve clinical outcomes. Compared with the placebo, patients receiving omega-3 fatty acids achieved significantly better results in several domains: the number of delirium days was significantly lower in the omega-3 group (2.83 ± 1.98 vs. 3.55 ± 2.02; *p* = 0.010), duration of mechanical ventilation was shorter (8.89 ± 5.29 vs. 11.13 ± 6.62 days; *p* = 0.008), and ICU length of stay was reduced (12.23 ± 5.11 vs. 14.91 ± 6.02 days; *p* = 0.001). However, no significant differences were observed in mortality or haloperidol administration. The study provides an initial rationale for further investigation into the effects of omega-3 fatty acids on delirium incidence in the ICU.

During preparation of the present materials, we identified studies that may support the findings reported by Wang et al. However, due to the fact that these are not clinical data, we did not analyze them in our review (Zhu et al. [40] and Yang et al. [41]).

### 3.8. Delayed Gastric Emptying

Delayed gastric emptying (DGE) is a common problem in the intensive care unit (ICU). It is estimated that gastrointestinal dysfunction may affect up to 60% of ICU patients [42] and that, in approximately 30% of patients in whom enteral feeding is attempted, the feeding route must be changed due to intolerance [43,44]. This issue is highly clinically relevant as it prolongs hospitalization and is associated with increased patient mortality [45].

According to the ESPEN 2023 guidelines, in cases of significant impairment of upper gastrointestinal motility, treatment involves pharmacotherapy or delivery of nutrition post-pylorically [14]. In ICU practice, pharmacological management of gastroparesis primarily includes erythromycin and metoclopramide. Both agents accelerate gastric emptying in critically ill patients. Because of its reportedly higher efficacy, erythromycin is considered the first-line agent in the ESPEN recommendations [14]. Combined use of both drugs may also be beneficial as they act through synergistic mechanisms—an effect supported by the study by Hersch et al. [46]. However, limitations of these therapies include potential adverse effects and the rapid development of tachyphylaxis.

A potential approach to mitigate or reduce the risk of delayed gastric emptying may involve the use of injectable lipid emulsions. Although further clinical studies are required, results published by Siginig Jing et al. evaluating the effect of perioperative administration of a fish oil-based injectable lipid emulsion (a source of omega-3 fatty acids) on postoperative gastric emptying after Roux-en-Y reconstruction for gastric cancer are encouraging [47]. In their retrospective cohort study of 210 patients undergoing partial gastrectomy with Roux-en-Y reconstruction, fish oil injectable lipid emulsion administration was associated with a 71% reduction in the incidence of DGE (4.3% vs. 15.5%; *p* = 0.005) along with faster symptom resolution, reflected by a 0.21-point improvement in the Gastroparesis Cardinal Symptom Index (GCSI) (95% CI: 0.02–0.4; *p* = 0.005).

A shorter median postoperative hospital stay was observed as well (10 vs. 13 days; *p* < 0.001).

### 3.9. COVID-19

COVID-19 is caused by infection with SARS-CoV 2, which triggers an immune response leading to pulmonary inflammation. In severe cases, a systemic hyperinflammatory reaction may develop, resulting in acute respiratory distress syndrome (ARDS) and organ complications. This uncontrolled inflammatory response is characteristic of cytokine release syndrome, involving increased concentrations of pro-inflammatory cytokines such as IL-6, IL-8, IL-17, IL-1β, and TNF-α. In severe COVID-19, an “eicosanoid storm” may also occur, driven by increased production of pro-inflammatory lipid mediators derived from arachidonic acid (an omega-6 PUFA), including prostanoids (prostaglandins and thromboxane) generated via the cyclooxygenase (COX) pathway and leukotrienes produced via the 5-lipoxygenase pathway. The excessive and unresolved inflammation observed in severe COVID-19 represents a classic example of impaired resolution of inflammation. Therefore, it has been hypothesized that omega-3 fatty acids, by supporting the formation of specialized pro-resolving mediators (SPMs), may promote inflammatory resolution.

The potential benefit of omega-3 fatty acids in patients with COVID-19 was evaluated by Arnadottir et al. [48] in the COVID-Omega-F trial (Resolving Inflammatory Storm in COVID-19 Patients by Omega-3 PUFA), a randomized, placebo-controlled, single-blinded study assessing the potential of intravenous omega-3 supplementation in the treatment of COVID-19. Hospitalized patients (n = 22) received for 5 days either 2 mL/kg placebo (0.9% NaCl) or a fish oil emulsion (Omegaven^®^, 10 g/100 mL). Fish oil therapy was associated with favorable modulation of the immune response; specifically, a significant reduction in the neutrophil-to-lymphocyte ratio was observed in the omega-3 group. The mean increase in lymphocyte count was 0.4 ± 0.08 × 10^9^/L in the intervention group versus 0.03 ± 0.16 × 10^9^/L in the placebo group (*p* = 0.03). No significant changes were found in total leukocyte count or in neutrophil and monocyte counts. CRP decreased from 60 (20–75) to 19 (3.8–27) mg/L after omega-3 administration and from 65 (40–92) to 32 (24–40) mg/L after the placebo (*p* = 0.08); however, these changes did not reach statistical significance. The intervention was also associated with increased levels of SPM precursors, reduced pro-inflammatory lipid mediators, decreased immunothrombosis, and enhanced phagocytosis in patients receiving omega-3 fatty acids. Based on these findings, the authors reported a beneficial immunological response following intravenous omega-3 administration in COVID-19 patients while emphasizing important study limitations, most notably the small sample size and the need for further research to confirm these effects.

Potential benefits were also reported by Berlan et al. [49] in a randomized, double-blind, single-center clinical trial (Omegaven + Smoflipid) evaluating the impact of omega-3 fatty-acid supplementation (Omegaven^®^ at 0.1 or 0.2 g/kg/day) added to a standard fish oil-containing injectable lipid emulsion (Smoflipid^®^) in COVID-19 patients requiring parenteral nutrition. The primary endpoint was the change in CRP and IL-6 over 4–9 days of therapy. The applied interventions supported the antithrombotic and pro-resolving properties of omega-3 fatty acids. The study confirmed favorable regulation of lipid mediators, including SPMs as key elements of inflammatory resolution. In addition, the 0.1 g/kg/day dose appeared to induce mainly immunomodulatory effects (CRP, IL-6, CXCL10), whereas the 0.2 g/kg/day dose was associated primarily with clinical effects, as this group demonstrated shorter hospital and ICU length of stay.

Similar positive conclusions regarding omega-3 use in patients with COVID-19 were drawn by Yue et al. [50], who conducted a randomized meta-analysis of available studies and ultimately included six trials involving 273 patients. Their results indicated that omega-3 fatty-acid administration was associated with reduced all-cause mortality among patients hospitalized due to COVID-19 (RR = 0.76; 95% CI: 0.61–0.93; *p* = 0.010).

Overall, the findings from clinical trials and meta-analyses suggest that omega-3 fatty acids may elicit a meaningful response supporting inflammatory resolution and exert anti-inflammatory effects in severe COVID-19. Nevertheless, larger, well-designed studies are still required to confirm these observations.

## 4. Conclusions

Omega-3 fatty acids (EPA and DHA) in injectable lipid emulsions used for parenteral nutrition represent an intervention whose potential extends beyond energy provision. The altered fatty-acid profile of modern mixed-oil ILE affects cell membrane composition, intracellular signaling pathways, gene expression and, most importantly, lipid-mediator synthesis. EPA and DHA compete with arachidonic acid, thereby modulating eicosanoid production, and they serve as substrates for the generation of specialized pro-resolving mediators (SPMs), which enable the physiological resolution of inflammation.

Based on the evidence summarized above, fish oil-containing emulsions may provide clinical benefits in critically ill patients, particularly by reducing the risk of infections and sepsis and shortening the duration of hospitalization. Selected analyses have also reported shorter ICU length of stay and reduced duration of mechanical ventilation, which may be clinically and organizationally meaningful. In acute pancreatitis, reductions in inflammatory markers and favorable effects on the course of organ dysfunction have been observed, while in severe COVID-19, features of immunomodulation and a shift in lipid-mediator profiles toward pro-resolution have been reported. In addition, observational data suggest a potential reduction in delayed gastric emptying after surgery as well as a possible decrease in delirium risk in patients with sepsis.

Despite these encouraging findings, substantial heterogeneity remains across studies, including differences in patient populations, dosing regimens, omega-3 doses, and types of injectable lipid emulsions. Inconsistent results regarding mortality and methodological limitations in some trials indicate that further well-designed randomized clinical studies are needed. Such trials should allow more precise determination of optimal dosing and timing of administration and help identify the patients most likely to benefit from omega-3-enriched injectable lipid emulsions. In light of the available data, clinical practice increasingly supports a preference for mixed-oil ILE supplemented with fish oil as part of parenteral nutrition in critically ill patients, provided that safety principles, metabolic tolerance, and the overall energy balance are carefully considered.

## Data Availability

No new data were created or analyzed in this study.
